# What individual needs do family caregivers have in palliative home care and how are they supported? A qualitative study of a supportive intervention

**DOI:** 10.1007/s00520-024-08904-6

**Published:** 2024-10-18

**Authors:** Christiane Kreyer, Barbara Stecher, Sabine Pleschberger, Gail Ewing

**Affiliations:** 1 Department of Nursing Science and Gerontology, UMIT TIROL-Private University for Health Sciences and Health Technology, Hall in Tirol, Austria; 2 Centre for Public Health, Medical University of Vienna, Vienna, Austria; 3 Centre for Family Research, University of Cambridge, Cambridge, UK

**Keywords:** Palliative home care, Family caregivers, Carer Support Needs Assessment Tool Intervention (CSNAT-I), Caregiver support

## Abstract

**Purpose:**

Family caregivers (FCGs) play a pivotal role in supporting patients in palliative care at home. Person-centred support is crucial to prevent negative outcomes; therefore, evidence-based approaches such as the Carer Support Needs Assessment Tool Intervention (CSNAT-I) are promising. To understand more about the delivery of the intervention, the study focuses on documentation of CSNAT-I in practice in Austria to identify which support needs were discussed with the FCGs and the types of support delivered to meet these needs.

**Methods:**

A retrospective analysis of electronic records was conducted, focusing on documented entries related to the delivery of CSNAT-I over a 21-month period (Dec 2019 to Aug 2021). Both qualitative and quantitative methods were employed for data analysis.

**Results:**

The analysis identified a wide spectrum of FCG support needs, categorised into enabling domains related to caregiving for the patient and direct support needs concerning FCGs’ own health and well-being. The most frequently documented support needs included ‘having time for oneself in the day’ and ‘dealing with feelings and worries’, highlighting the challenges FCGs face in balancing caregiving responsibilities with personal life. Supportive input encompassed advice and information, counselling, education and training, coordination and arrangement, and signposting and referral.

**Conclusion:**

The study stresses the importance of addressing both practical and psychosocial aspects of caregiving, utilising a person-centred approach. Nurses provided comprehensive support mostly directly delivered during their contact with FCGs. CSNAT-I demonstrated flexibility, accommodating the diverse needs of FCGs in different situations, and may contribute to a more supportive care environment.

## Introduction

Family caregivers (FCGs) play a key role in supporting patients in palliative care and enabling dying at home [1, 2]. Several studies show that the FCG role is demanding and may have negative effects on health and wellbeing [3–5]. FCGs have a dual role, as co-workers and co-clients, and may have support needs in both dimensions [6, 7]. FCGs in palliative care frequently report unmet support needs, particularly highlighting the neglect of their psychosocial and emotional well-being [7–9]. Research recognises the need for person-centred support for FCGs to prevent negative outcomes [10]. Given the impending shortage of nursing personnel, ensuring comprehensive support for FCGs becomes even more crucial.

Palliative home care services provide support to patients and FCGs by providing competent care and being present [11]. Supporting FCGs stands as an inherent conceptual component of hospice and palliative care [12]. However, what precisely falls under its scope, how the need for support is determined, and the specific ways in which FCGs are supported often remain undefined, and nursing concepts for this purpose are lacking [13–15]. FCGs’ support needs are usually not systematically assessed [16]. Instead, healthcare professionals frequently conduct informal, ad hoc assessments of FCG needs [15]. Additionally, the support needs and related supportive provision are often not documented [16]. Especially the separate support needs of family members are often overlooked [7], partly because interactions with FCGs typically occur in the presence of the ill person. Nurses might be concerned that focusing on FCG needs could increase their workload and create unrealistic expectations about the support they can provide [17].

**Fig. 1:**
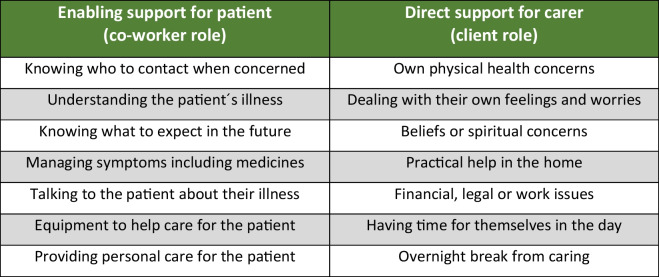
The 14 domains of support needs included on the CSNAT (v2.0)

**Fig. 2:**
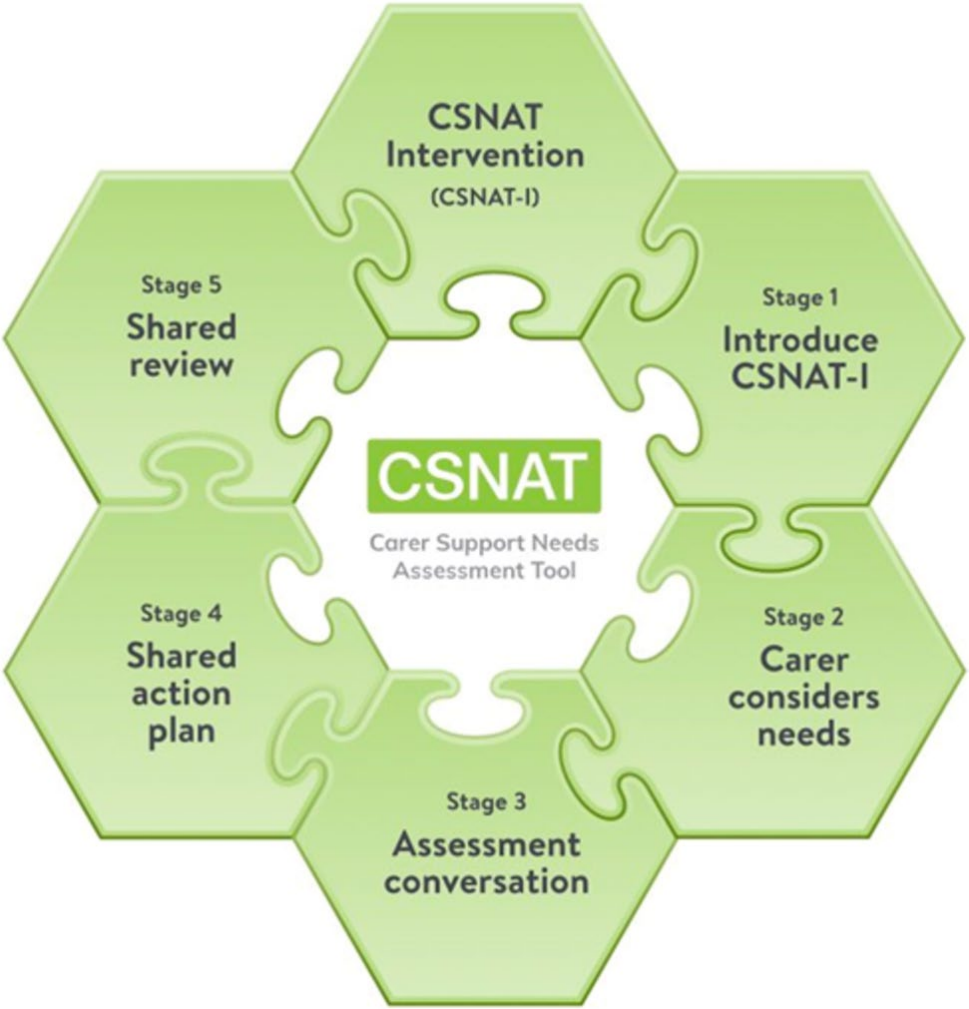
The five-stage person-centred process used with CSNAT-I. Delivery of the intervention begins with (1) introduction of CSNAT-I to the FCG, (2) FCGs are given time to reflect and self-complete the-tool itself to identify the domains with which they need more support, (3) an assessment conversation takes place between the FCG and the nurse where the domains prioritised for discussion by the FCG are further explored to identify the FCGs individual needs within the prioritised domains, (4) negotiation of a shared action plan to meet the identified needs, and (5) a shared review of the results of this process. The individual needs of the FCGs identified through this process and the agreed actions to meet these needs are recorded on the CSNAT-I Support Plan, which includes the relevant support domain prioritised, the support needs discussed, and the corresponding support actions

The Carer Support Needs Assessment Tool Intervention (CSNAT-I), developed initially for supporting FCGs providing end-of-life care at home, is a person-centred intervention, facilitated by healthcare professionals but led by the FCG [7, 18]. This means that the initiative remains centred on the needs and preferences of the caregivers themselves. Acknowledging the complexity of the FCG role, this model enables a tailored approach to address individual support needs. It has proven to be effective with reduction in FCG strain [19], in distress [20], improved outcomes in bereavement [21], and increased preparedness for caregiving [22]. In the German context, the intervention is known as KOMMA-approach [23, 24]. CSNAT-I is underpinned by an evidence-based self-assessment tool (the CSNAT) comprising 14 domains (broad areas of support needs), falling into two groupings: (1) support needs FCGs may have to be able to care for the patient in their co-worker role (the enabling domains) and (2) support needs concerning their own situation, health and well-being in their co-client role (see Fig. 1). The intervention, CSNAT-I, is then delivered using a five-stage person-centred process [18] (see Fig. 2).

CSNAT-I is a promising supportive intervention containing assessment, tailored support, and documentation with proven effects. However, the specific content discussed with the FCGs and how nurses address FCG needs when CSNAT-I is delivered in routine practice have not been presented thus far. Therefore, to understand more about the delivery of the intervention and to further inform training in its use, the study focuses on documentation of CSNAT-I in practice to identify which FCG support needs were discussed during the assessment conversation with the FCGs and the types of support delivered to meet these needs.

## Methods

### Context

This study draws on an implementation study of CSNAT-I conducted in the Tyrol region of Austria, which has a population of approximately 755,000 inhabitants. Between 2019 and 2022, specialised palliative home care teams (SPHC) integrated CSNAT-I into their practices, aiming to incorporate it into their daily routine activities. The initiative involved seven SPHC teams, encompassing approximately 50 staff members, mostly specialised nurses, responsible for providing care to adult patients in palliative care and their families. In addition to nurses, SPHC teams in Austria typically also include physicians and sometimes social workers, and psychologists. As the use of CSNAT-I was a change from usual practice, the research team provided both initial and ongoing training and support for the staff in delivery of the intervention. Within each team, designated champions facilitated the integration and utilisation of the CSNAT-I. Typically, the SPHC-team introduced CSNAT-I to the FCG at the onset of care and offered an assessment conversation. These conversations were conducted either in the patients’ homes or by telephone, a practice influenced by the constraints imposed by the COVID-19 pandemic.

In Austria, it is common for all care to be documented in the patient’s record, including supportive care for FCGs. All participating teams used the same electronic record system, named PalliDoc®. A dedicated section within PalliDoc® was created collaboratively with the teams to accommodate the key elements of the CSNAT-I Support plan which documents the use of the intervention. This section included a drop-down menu that facilitated automatic selection of the 14 CSNAT domains, which then linked to a text field for entries related to the discussed support needs and corresponding supportive input put in place to meet the identified support needs. The nurses were responsible for documenting the FCGs’ support needs and the actions taken to address them.

### Study design

A retrospective analysis of the electronic records was conducted, using both qualitative and quantitative methods to analyse the documentation of FCG assessment and support when CSNAT-I was delivered.

### Ethical issues

Ethical approval was received by the Research Committee for Scientific Ethical Questions (RCSEQ) body of the university of UMIT TIROL (statement of 23.4.2021, no. 2746). The management were informed about the procedure before the respective team was included and gave their written consent to the data collection. The regional palliative care coordinator responsible for the quality of care had access to anonymised extracts from the electronic records for quality assurance purposes.

### Sampling

The palliative care coordinator provided the research team with all entries made in the newly created text field within the implementation period from December 2019 to August 2021 (21 months). The data submitted were fully anonymised, i.e. the researchers had no information about the families receiving palliative care or the teams providing care. The research team selected all entries in which CSNAT domains were checked (via a drop-down menu), indicating that a CSNAT conversation had taken place about those domains.

### Data collection

We developed a form to extract data based on the CSNAT-I Support Plan: (i) the documented CSNAT domains prioritised by the FCGs, (ii) documented support needs discussed with the FCGs, and (iii) corresponding documented supportive input/action plans to address identified support needs.

### Data analysis

We used qualitative and quantitative methods to analyse data. We conducted a qualitative content analysis of the data (Kuckartz & Rädiker, 2022). Data were coded deductively using the framework of the 14 domains of the CSNAT and the categories *support needs*, *supportive input*, and *no entry*. Subsequently, subcategories were formed inductively for each category with the aim of summarising the FCGs unmet needs and categories of supportive input. MAXQDA was used to facilitate data management. Descriptive statistics were used to analyse the number of entries and the frequency of particular CSNAT domains, using Microsoft Excel.

### Trustworthiness and data quality

To ensure the trustworthiness and quality of the data, we employed several measures. The electronic records were maintained in the PalliDoc® system, a standardised and reputable platform, ensuring consistent and systematic data recording across all participating teams. A structured form was developed to extract data based on the CSNAT-I Support Plan. This form facilitated the consistent capture of information on CSNAT domains, support needs, and supportive input/action plans. All nurses involved in CSNAT-I assessments received comprehensive training on both the intervention and the PalliDoc® system. A standardised documentation protocol, including clear guidelines for using drop-down menus and text fields, was established to ensure uniform data recording. Regular meetings with team representatives were held to discuss and address any issues related to documentation and data consistency. These steps collectively contributed to the reliability and validity of the data used in this study.

### Research team and reflexivity

BS, a nursing bachelor student with a special interest in nursing research, performed the initial coding. CK, a nurse and experienced qualitative researcher, and BS then developed together inductive categories and a final coding system. After discussion and agreement within the research team, BS mapped the documentation entries inductively. Verification of the mapping process was conducted by CK. Finally, content and analysis were discussed and adapted within the research team.

## Results

**Fig. 3:**
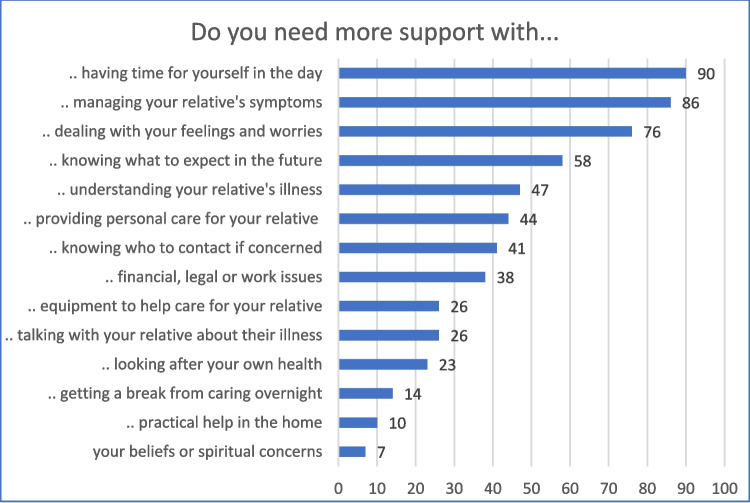
Frequency of documented CSNAT-I domains of caregiver support needs (*n* = 586)

Individual CSNAT-I assessment conversations were conducted with 484 family caregivers and subsequently documented in the electronic records. These covered 586 assigned CSNAT domains. Their frequency is shown in Fig. 3. The following domains among FCG support needs were documented most often: ‘having time for yourself in the day’, ‘managing your relative’s symptoms’, and ‘dealing with your feelings and worries’.

Eleven percent of the entries documented FCG needs discussed but no corresponding actions to address these needs. Domains in which no corresponding supportive input was documented most frequently were ‘dealing with your feelings and worries’ (*n* = 14) and ‘having time for yourself in the day’ (*n* = 13). There were also shortcomings in documenting FCG support in terms of wording, completeness, and recording of the person-centred process.

## Domains of support needs

In the following, we provide an overview of the identified domains of support needs, firstly the enabling domains, then the direct support domains, sorted by frequency of documentation.

### Enabling domains—support needs when caring for the patient

FCGs had a range of support needs to enable them to care for the patient in their role as co-worker. Table 1 outlines documented support needs across different domains of the CSNAT.

**Table 1:** Enabling domains - support needs & support measures

CSNAT-domain (main category)	Subcategories	Caregivers’ unmet needs	Supportive input/action plan
**Managing your relatives symptoms and medication (86 entries)**	Dealing with symptoms	Pain Management (patient does not call attention to pain; confusing expression of pain; insufficient pain relief) Swallowing problems Fear of choking Unstable trajectory Problems with eating Sleep disorder Patient‘s behaviour (reacts harshly; does not speak; neglects side-effects) Worries about missing something	Advice & information – Medication and their administration (indication, effects and side-effects, medication plan, medication on demand, monitoring medication, discontinuing medication) – Emergency behaviour (emergency plan, emergency kit, administering emergency medication, when referring to hospital, measures against panic attacks) – Symptom control (Pain, restlessness, appetite and nutrition, oral care, dying process, symptom control via body language, tips for calming down, breathing exercises) – Technical information Coordination & arrangement – Regular phone contact with FCG – Planning pro-active asking for uncertainty – Establishing emergency management – Establishing PCA pump or other devices – Providing medication plan – Preparing medication Education and training – Administering medication – Performing subcutaneous injection – Handling PCA pump, port system – Acting in case of emergency Signposting & referral – Appointment with doctor, service provider
	Dealing with medications	Prescribed medication not sufficient Patient or FCG reject medication Problems taking medication Uncertainty preparing and administering medication More information and training required Support through health service required	
	Emergency management	Dealing with panic and fear attacks Dealing with bleeding Patient isn´t able to speak	
	Technical problems	Pump handling Handling devices	
**Knowing what to expect in the future (58 entries)**	Fears & challenges	Fears about future development Fears about what will happen Questions about what will happen Patient refuses options, FCG fears what will happen Patient don’t want to live anymore, spouse very sad Wonders, if she will manage everything	Advice & information – Additional services & help – Illness trajectory, symptoms expected – Dying & death: signs, duration, rituals – What to do after death – Symptoms, eating/drinking, breathing, care, emergency Counselling – Reassuring – Fears and uncertainties Coordination & arrangement – Emergency plan – Time resources for counselling Signposting & referral – Live-in care – Nursing home
	Dying & death	Questions, when death is to be expected Questions, how dying is like Knowledge, which symptoms will be expected	
	Specific questions	Wants to be very well prepared for future Wants to get to know preventative measures Wishes that patient gets to know inpatient palliative care unit Needs more information on very fast deterioration	
**Understanding your relative´s illness (47 entries)**	Knowledge about the illness	Wants to know how things are going with his wife, why she is no longer receiving treatment, where the metastases are Requires knowledge about illness trajectory Does not feel sufficiently informed in general	Advice & information – Limited lifetime, illness trajectory, symptoms – Writing down questions Counselling – Need for talk with doctor – Commitment for support Coordination & arrangement – Round table with family & professionals Signposting & referral – Doctors, hospital
**Knowing who to contact if you are concerned about your relative (41 entries)**	Availability of health care professionals	Availability during night and weekends Availability in case of emergency, fall, deterioration Availability of family doctor or PHC team Burden, nobody was available in the evening	Advice & information – PHC Team availability 24/7 – Emergency phone numbers – Responsibilities of care providers – Using baby phone Coordination & arrangement – Additional appointments – Forming formal & informal network – Emergency bracelet
	Finding the right support	Whom to ask for specific medical and caring questions	
**Providing personal care for your relative (44 entries)**	Difficulties, ambiguities within the family & others involved	Worries about increasing caring needs Patient very self-determining Difficulties performing caring tasks Cooperation with community care, doctor Patients only wants to be cared for by spouse Grandson doesn’t want to perform caring tasks	Advice & information – Suggesting further services, medication, equipment Counselling – Fears, uncertainties on bodily caring – Communication problems Coordination & arrangement – Equipment, training – Coordinating various providers Education/Training FCG & other HCP – Oral care – Incontinence care – Mobilisation – Stoma, port, wound care – Handling equipment Signposting & referral – Care & nursing services
	Burden, overload	Burdened with increasing caring needs Need for assistance in personal care tasks FCG is moving away	
	Particular caring tasks & equipment	Questions about personal care, skin care, mobilization Problems changing incontinence pants, handling body chair Handling diarrhoea at night	
**Equipment to care for your relative (26 entries)**	Obtaining equipment	Talking about thoughts obtaining equipment Discussing need of emergency contact button, nursing bed, toilet chair, roller walker	Advice & information – Equipment obtainment, use – Payment – Clarifying who organises, who gets it Coordination & arrangement – Equipment – Training
	Accessing equipment	Knowing were to get equipment Financing of equipment Problems in accessing equipment	
	Use of equipment	Uncertainty and questions using equipment	
**Talking with your relative about his or her illness (26 entries)**	Illness knowledge	Patient doesn’t know much about prognosis 2 of 3 children don’t know about the seriousness of the illness	Advice & information – Developing family plan – Psychosocial support – Offers to talk with FCG, patient, children – Round table Counselling – Listening, reassuring, relieving – Allowing emotions – Dying & death – Talking with family Signposting & referral – Doctors, hospital
	Illness perception	Patient doesn’t accept illness Patient hopes for healing, FCG has difficulties talking about dying Patient thinks he can do everything by himself Difficulties to accept patient´s treatment decision Change of character causes conflicts	
	Arranging conversations	Wish for a conversation between doctor and patient Seeking contact to a self-help group	
	Talking about dying & death	Patient wants to talk, sons not Daughter does not dare to talk about, wishes to get help from HCP Feels alone choosing treatment options Does not know how to respond to patient statements Problems to talk about deterioration Child cries, doesn’t want to talk with patient about illness Patient talks about dying, FCG can’t cope	

The need for more support was most commonly recorded for ‘Managing symptoms including medicines’. Many entries focused on handling symptoms, administering medication, and emergency management. FCGs’ support needs varied widely, including issues of the management of pain, swallowing, eating, sleep, behavioural issues, and fluctuations in the patient’s condition. Concerning medication, issues ranged from prescription to administration, including challenges with patient ability or refusal to take medication, alongside general fears of overseeing something important.

With ‘Knowing what to expect in the future’, FCGs discussed fears and challenges regarding the progression of the patient’s illness. More support was also needed when FCGs had to deal with patient behaviours such as expressing a wish not to continue living. Several entries related directly to death and dying, including when death may occur, the process of dying, and symptoms to expect during this time. Similar issues of disease progression, life expectancy, and symptoms were also identified by FCGs seeking more support with ‘Understanding your relative’s illness’ as well as knowledge deficits regarding the patient’s illness itself.

‘Providing personal care’ comprised not only questions concerning particular caring tasks but also challenges in performing these within the network of the patient, family, and professionals. A sense of burden was expressed by FCGs due to the feeling that caregiving tasks became overwhelming. FCGs had support needs in not only obtaining and accessing equipment to care for the patient but also in knowing how to use it. Accessibility of professionals during nights and weekends was needed by FCGs, as well as who to contact in case of emergencies, such as a fall or worsening symptoms.

Finally, FCGs encountered difficulties in ‘Talking about the illness’ when the patient or other family members lacked prognosis awareness. Also, support needs concerning illness perception occurred, with some patients refusing to acknowledge the illness severity and instead hoped for recovery. Disagreements over decisions caused further FCG needs. Discussing death and dying posed challenges, as individuals struggled to broach the subject or accept mortality. FCGs also asked for facilitating conversations with the patients on these topics.

### Direct support needs—FCGs’ own health and well-being needs

The entries also revealed the extent of direct support needs, which means support required to preserve their own health and well-being in their role as clients. Table 2 shows the documented direct support needs in the different domains of the CSNAT.

**Table 2:** Direct support needs—FCGs’ own health and well-being needs and support measures

CSNAT-domain (main category)	Subcategories	Caregivers’ unmet needs	Supportive input/action plan
**Having time for yourself in the day (90 entries)**	Dealing with caregiver burden	Feels burdened Difficulties to reconcile things Only very few reserves left Patient is demanding, FCG is doing household chores and care alone Son is alone most of the time Patient does not accept assistance	Advice and information – Additional support (volunteers; community nursing; psychologist; social work) Counselling – Resources and stress factors, limits – Coping strategies – Taking breaks, walks, holidays – Support via family, friends, neighbours – Reorganising daily schedule – Talk with patient, family Coordination and arrangement – Additional care Signposting and referral – Hospice volunteer service – Care and social services
	24/7 care	Caring 24/7 for the patient, no time for own family Does not want to leave patient alone	
	Fears leaving patient alone	Fear of fall Fear of convulsion Fear that patient would not eat when she is not there Fear of COVID-infection Assistance available, cannot let go Cannot confine patient to anyone	
	Communication within family	Nurse shall talk to patient, that FCG needs breaks Needs break, patient should be admitted to hospital Does not want to burden adult children	
	Performing activities	Wants two short breaks a day Wants to do more activities Wants to take walks Asks, if situation allows to take a break	
**Dealing with your feelings and worries (76 entries)**	Family conflicts	Family history, difficult family situation Communication problems Additional burdens within the family Conflict with patient	Advice and information – Psycho-social services, psycho-oncology – Seeking help from family, friends – Reassuring help from PHC team – Hospice volunteer service Counselling – Listening, reassuring, relieving – Own needs, resources, limits
	Dying and death	Talking about dying and death Dying in dignity and peace Worries about drinking and eating Experiences with undignified death	
**Dealing with your feelings and worries (continued) (76 entries)**	Burdening situations	Patient angry, aggressive Patient accuses helper Rapid deterioration Tumour recurrence Wellbeing of other family members Feels not to be taken seriously by healthcare professionals Sleep disorder Own eye surgery, worries about seeing good enough for caring Received blames Overseeing serious symptoms Corona pandemic	– Allowing emotions – Dying and death – Talking with family Coordination and arrangement – Further telephone calls and individual Counselling – Clarifying conversation with a person – Videoconference with daughter Signposting and referral – Psycho-social services, psycho-oncology – Hospice volunteer service
	Inner personal conflicts	Talking about own fears and worries Wants to enable dying at home, worries to be overburdened Wants to be a good carer, experiences too much responsibility Wants to be present, but withdraws herself Needs somebody to talk, but social network missing Worries, if delineation is right strategy in her situation Feels guilty because she thinks dying would be a salvation	
**Your financial, legal or work issues (38 entries)**	Care allowance	Notification missing Missing documents Care level assessed too low, needs to be increased	Advice and information – Care allowance, hospice leave – Legal, financial support – Financing options Counselling – Working situation Signposting and referral – Social worker – Case and care management
	Work issues	On leave until January, what happens then? Concerns about late payment for hospice leave Possibilities to reduce working hours Wish to discuss if care leave or quit job is better	
	Financial issues	Money for caring aids, bathroom remodelling Information about subsidy application live-in care Co-insured with patient—what happens after death? Do not know password of savings book any more Care financially difficult to manage	
**Looking after your own health (23 entries)**	Individual health problems impacting on caring tasks	Needs surgery Needs rehabilitation after health issues Needs medical appointment Suffers from epilepsy, might be a challenge Suffers from stomach pain Feels physically exhausted Has problems with thyroid Gets increasingly sad Cannot lift heavy after disc surgery Has neurological problems	Advice and information – Respite care – Inpatient care – Hospice volunteer service – Psychological help – Creating care network Counselling – Importance of own health – Resources and limits – Attending medical appointments – Talking with family
	Health related questions	Worries about genetic disposition Use of sedatives for sleeping	
**Getting a break from caring overnight (14 entries)**	Availability and worries	Does not know whom to call during night hours Burden by sleep deficiency Does not sleep well, looks several times after patient Worries, patient will stand up during the night Worries, not to hear patient during sleep	Advice and information – Live-in care – PHC Team availability 24/7 – Sensor pad – Involving family Counselling – Reassuring
**Practical help around the home and elsewhere (10 entries)**	Household chores	Need for help in the household (not specified)	Advice and information – Involving family, neighbours Coordination and arrangement – Household service
**Your beliefs or spiritual concerns (7 entries)**	Believing and pastoral care	Irritation, because patient asks for a priest, without believing Irritation, because patient do not want a priest, despite believing Thoughts about life after death	Advice and information – Availability pastoral care, priest – Anointing of the Sick Counselling – Faith, belief – Expectation, wishes, concerns

‘Having time for yourself’ was the most common direct support domain as well as the most common support domain in general. FCGs expressed feelings of burden due to diminishing personal resources and being solely responsible for caregiving, but also that the patient was demanding and refused alternative support. Balancing other responsibilities, like their own family, alongside 24/7 caregiving responsibilities was challenging. Various fears concerning the patient made it impossible to take time for themselves.

Many ‘Feelings and worries’ were raised by FCGs. These included family conflicts, communication issues and concerns for other family members. Emotions and burdens were articulated, particularly concerning the patient, such as aggression, refusal of help, or rapid deterioration. This also included discussing death and dying and ensuring the patient’s dignity. But FCGs also took opportunity to discuss inner personal conflicts, experiences of insomnia, and feeling unheard.

Financial issues were raised by FCGs including queries regarding caregiving allowance, inadequate allowance levels, and money worries related to providing care. There were also some work-related concerns such as reducing working hours or applying for caregiving leave.

FCGs raised fewer support needs in the remaining four ‘direct’ domains. A range of different individual health issues impacted on caregiving, such as experiencing different symptoms and requiring treatments or medications themselves. Unlike having time for self in the day, fewer FCGs had support needs with overnight caring though there were issues around availability of help and worries about the patient’s safety. Overall, FCGs had fewest unmet support needs related to ‘Practical help’ as well as ‘Beliefs and spiritual concerns’.

## Supportive input

We identified five types of supportive input in the documentation provided by the nurses (see Tables 1 and 2). Most of the input was directly delivered by the nurses during the assessment conversation/action planning stages of CSNAT-I.

*Advice and information* encompassed the provision of guidance, recommendations, and knowledge aimed at aiding the caregiving process. Documented advice involved offering suggestions, strategies, or instructions regarding health-related decisions, treatment options, and self-care practices. This encompassed available support resources, such as respite or inpatient care, additional services, medication and equipment, legal and financial support options, and finally, on creating a care network involving family and friends. Information referred to the provision of factual data, explanations, and informational materials to enhance understanding and awareness of palliative care issues. Nurses informed FCGs about various aspects of the health condition and potential complications concerning the illness trajectory, expected symptoms, death and dying, emergency management, symptom control, and the responsibilities of involved healthcare providers.

*Counselling*, in the context of this study, refers to the process of providing emotional and psychosocial nursing support to FCGs aiming at helping individuals cope with various issues, make decisions, and manage their feelings and concerns related to palliative care at home. Nurses documented providing emotional support and promoting effective communication between FCGs, patients, and their families. This is reflected in entries where fears and uncertainties are addressed and reassurance and understanding were offered. Nurses fostered open and honest conversations about dying and death and supported FCGs to have these difficult conversations and address concerns and wishes regarding treatment preferences, spiritual beliefs, and final arrangements. They also promoted self-care practices such as taking breaks, seeking social support, and engaging in stress-relieving activities. Additionally, FCGs were encouraged to utilise support networks, including friends, family, and community resources, to lessen the burden of caregiving.

*Education and training* involved providing knowledge, skills, and resources aimed at empowering FCGs with the necessary tools and information to deliver quality care, maintain their well-being, and enhance the overall caregiving experience. FCG education focused on increasing FCGs’ understanding of the specific health condition or challenges faced by their care recipient. FCG training involved teaching practical caregiving skills and techniques to help FCGs perform their duties safely and effectively. Training covered a range of topics, including oral care, incontinence care, mobilisation, stoma and wound care, port catheter care, and equipment handling.

*Coordination and arrangement* refers to the organisation and management of various aspects of care to ensure smooth and efficient delivery of services. Activities included coordinating various providers, collaborating within the formal and informal care network, organising additional care, and facilitating roundtable discussions. Arrangements involved logistical aspects such as creating an emergency plan, organising equipment, providing training opportunities, household services, or arranging additional appointments.

*Signposting and referral* involved guiding patients to appropriate resources, services, or healthcare professionals to address their specific needs and concerns. Signposting referred to the process of providing clear directions, information, or guidance to FCGs regarding available support services, community resources, or relevant healthcare professionals, leaving them to make contact themselves. Referral involved formally directing FCGs, with their consent, to specialised healthcare providers or services for further support. This encompassed available services such as doctors, hospitals, nursing homes, care services, psycho-social services, case and care management, and hospice volunteer services.

## Discussion

This study examines the documentation of FCG support needs assessment and subsequent supportive input delivered during the implementation of the Carer Support Needs Assessment Tool Intervention (CSNAT-I) within specialised palliative home care teams (SPHC) in Austria. The findings provide new data about the use of the CSNAT-I in palliative home care practice and give valuable insights into the specific support needs experienced by FCGs and the types of supportive input delivered by nurses to meet these needs.

The documented entries reveal a spectrum of support needs experienced by FCGs, delineated into two overarching categories: enabling domains related to caring for the patient and direct support needs concerning the FCGs’ own health and well-being [18]. Within these categories, FCGs articulated concerns ranging from managing symptoms and medicines, understanding the patient’s illness trajectory, and navigating complex emotional and practical challenges. The prevalence of support needs related to ‘Having time for oneself in the day’ and ‘Dealing with feelings and worries’ emphasises the challenges FCGs face in balancing caregiving responsibilities with personal life and managing their emotions and worries. A UK study on the suitability of the CSNAT-I for FCGs of people with moto neurone disease uncovered similar issues [25]. These data on the use of the intervention underscore the critical importance of addressing not only the practical aspects of caregiving but also the psychosocial and emotional dimensions. The entries also indicate that FCGs used the offered assessment conversation to discuss their personal needs.

Interestingly, several individual support needs came up under different domains, e.g. dying and death, caregiver burden, fears, life expectancy, or uncertainties in symptom management. These cross-cutting themes show that there is no ‘correct space’ for bringing up a certain issue but emphasize the importance of the CSNAT-I as a conversation starter. The crucial point seems to be the process of self-reflection and communication, which underlines the need for a person-centred approach to FCG support in nursing [15]. However, the results also show that, despite being a structured approach, the CSNAT-I demonstrates flexibility, accommodating the diverse needs of FCGs in different situations.

The study highlights the diverse approaches employed by healthcare professionals to address FCGs’ support needs in their routine practice. We discerned five types of supportive input provided during delivery of CSNAT-I, including advice and information, counselling, education and training, coordination and arrangement, and signposting and referral. These interventions encompass a holistic approach aimed at equipping FCGs with the necessary knowledge, skills, emotional support, and practical resources to navigate the challenges of palliative care at home effectively. Nurses may worry that addressing FCG needs could add to their workload and raise unrealistic expectations about the support they can provide [17]. In contrast, most of the identified nursing activities in this study were directly delivered during the contact with the FCG. This was also the case in the study by Lund et al. [26]. Nurses are mostly unaware of the power of directly delivered nursing interventions like listening, encouraging, informing, and giving advice. In general, nursing work entails numerous unrecognised aspects [27]. Whilst the term ‘counselling’ may typically suggest psychological expertise, in this study, it is used to describe the supportive role that nurses play in addressing emotional needs and facilitating communication, which is an integral part of their palliative care practice.

Finally, nurses have been shown to report their interventions inadequately and perform many more caring tasks than they document [16]. They mostly document biomedical issues and inadequately record psychosocial, social, cultural, and spiritual aspects of care [16, 28]. Time for documenting tends to be overestimated [29]. Hardly any provided supportive interventions to FCGs are documented [16]. In contrast, our study underscores the informative value of comprehensively documenting FCG support, particularly related to identifying support needs FCGs have and the type of supportive input that can be provided for them in routine palliative care nursing practice.

Whilst implementation of evidence-based interventions is vital in nursing, CSNAT-I can represent a change from usual practice of FCG support that brings with it concerns about increased workload and fears of ‘opening a can of worms’ by asking FCGs about the support they need (17). The importance of this study is to allay such concerns in that the findings clearly show that the support needs that arise from using CSNAT-I can be addressed by palliative care nurses, mostly directly delivered during the contact with the FCG. As such, the study findings will provide a valuable additional resource for the CSNAT-I Training and Implementation Toolkit that is available to practitioners wishing to use the intervention in practice (https://arc-gm.nihr.ac.uk/training/register).

## Limitations

The use of electronic records from the CSNAT-I assessment conversations presents certain limitations. Specifically, the structured nature of the CSNAT (the tool itself) may constrain the breadth of information captured, focusing on predefined questions and domains. However, the CSNAT was developed through an extensive qualitative study involving 75 participants (7) and has been validated in several other studies (19–22), which suggests that the support needs of caregivers are comprehensively represented.

Despite these limitations, our study aimed to analyse the documentation of CSNAT-I in routine practice to gain insights into its practical application. By examining how FCG support needs were discussed and addressed through documented entries, we sought to understand the real-world delivery of the CSNAT-I intervention and identify areas for potential improvement in training and practice. The structured format of the CSNAT, whilst limiting the scope of data, provides a consistent basis for evaluating the intervention’s effectiveness and implementation. This focus on documentation allows us to explore the specific content discussed during CSNAT-I assessments and the types of support delivered, contributing valuable insights into the practical use of the tool in palliative care settings.

The study was conducted in one region in Austria and may be influenced by the organisation and structure of palliative home care in this region. Future research should incorporate qualitative interviews or focus groups with FCGs to provide a deeper understanding of their perspectives. Additionally, longitudinal studies are needed to assess the long-term impact of supportive interventions on FCGs.

## Conclusions

In conclusion, our study highlights the necessity of comprehensive support to address the multifaceted needs of FCGs in palliative home care. CSNAT-I is designed to identify and address different support needs, including those that may require additional involvement from physicians, psychologists, social workers, and other professionals. Whilst our study focuses on the nursing delivery of this intervention, it is important to recognise that effective palliative care necessitates an interprofessional approach to fully meet the needs of FCGs.

By providing insights into the specific domains of FCG support needs and the types of supportive input delivered, this research contributes to the professionalisation of FCG support in specialised palliative home care, as also Norinder et al. [30] have stated. Moving forward, efforts should be made to further integrate person-centred approaches like the CSNAT-I into routine care practices of palliative home care and to continuously evaluate and refine documentation processes to ensure comprehensive and effective support for FCGs.

## Data Availability

Data is provided within the manuscript.
